# Why are social sciences and humanities needed in the works of IPBES? A systematic review of the literature

**DOI:** 10.1080/13511610.2018.1443799

**Published:** 2018-03-01

**Authors:** Alice B. M. Vadrot, Aleksandar Rankovic, Renaud Lapeyre, Pierre-Marie Aubert, Yann Laurans

**Affiliations:** a Centre for Science and Policy, 10 Trumpington St, CambridgeCB2 1QA, UK; b Department of Political Science, University of Vienna, Ferstelgasse 5, 1090Vienna, Austria; c Institute for Sustainable Development and International Relations, Sciences Po, 27 rue Saint-Guillaume, 75007Paris, France

**Keywords:** IPBES, social sciences, interdisciplinarity, biodiversity, conservation, science-policy interface

## Abstract

Despite the increased attention, which has been given to the issue of involving knowledge and experts from the social sciences and humanities (SSH) into the products and works of the Intergovernmental Science-Policy Platform on Biodiversity and Ecosystem Services (IPBES), little is known on what the expectations towards the involvement of SSH in IPBES actually are. The aim of this paper is to close this gap by identifying the range of possible SSH contributions to IPBES that are expected in the literature, and discuss the inherent challenges of and concrete ways to realize these contributions in the particular institutional setting of IPBES. We address these two points by: *firstly*, assessing the literature dealing with IPBES and building a typology describing the main ways in which contributions from SSH to IPBES have been conceived between 2006 and 2017. We discuss these expected contributions in light of broader debates on the role of SSH in nature conservation and analyse some of the blind spots and selectivities in the perception of how SSH could substantially contribute to the works of IPBES. Then, *secondly*, by looking at one particular example, economics and its use in the first thematic assessment on pollinators, pollination and food production, we will concretely illustrate how works in a given discipline could contribute in many different and unprecedented ways to the works of IPBES and help identify paths for enhancing the conservation of biodiversity. *Finally*, we propose a range of practical recommendations as to how to increase the contribution of SSH in the works of IPBES.

## 1. Introduction

The creation of the Intergovernmental Science-Policy Platform on Biodiversity and Ecosystem Services (IPBES) has given fresh impetus to debates over the role of the social sciences and humanities (SSH) in nature conservation. Compared to the Intergovernmental Panel on Climate Change (IPCC) or to previous initiatives on biodiversity, such as the Global Biodiversity Assessment (GBA), the Millennium Ecosystem Assessment (MA) or the Global Biodiversity Outlook (GBO), IPBES aims to integrate experts from a wider range of scientific disciplines and knowledge backgrounds. The Panama resolution, adopted in 2012 by almost 100 countries, explicitly recognizes the need for “multidisciplinarity”, defined as

an approach that crosses many disciplinary boundaries, knowledge systems and approaches to create a holistic approach, focusing on complex problems that require expertise across two or more disciplines. Multidisciplinarity arises when scientists (including natural and social scientists), policy and technical experts, natural resource managers, other relevant knowledge holders and users, interact in an open discussion and dialogue giving consideration to each. (UNEP/IPBES.MI/2/9, p.17)

Particularly after the adoption of the conceptual framework by the IPBES plenary in 2013, the need for extending the relevant expertise to different disciplines from SSH became more apparent (Diaz et al. 2015; Joly 2014; Borie and Hulme 2015). The emphasis on the economic and non-economic value associated with biodiversity has anticipated the need for methodologies and concepts from a broader academic spectrum including e.g. economics, ethics political science, history and anthropology. Studying and understanding the direct and indirect drivers of biodiversity loss has been recognized together with the need to consider scholarly work on the political, socio-economic and legal conditions within which certain policy measures are developed and implemented. The importance of multidisciplinarity is being formulated by means of “[r]ecognizing, making visible, and respecting the diverse values at stake and addressing power relations through which these are expressed, are all needed in order to effectively and equitably bridge different value systems, eventually allowing processes of social learning” (Pascual et al. 2017, 11). Consequently, the IPBES approach has moved from ecosystem services to contributions introducing the concept of “nature’s contributions to people” (NCP) as a baseline for its global and regional assessments (Díaz et al. 2018). The NCP concept “[…] extends beyond the highly influential yet often contested notion of ecosystem services, incorporating a number of interdisciplinary insights and tools” (Díaz et al. 2018, 272).

In IPBES history, however, involving experts from SSH into IPBES working groups has quickly constituted a difficult task (e.g. Duraiappah and Rogers 2011; Vadrot 2011; Hulme et al. 2011; Vohland et al. 2011; Turnhout et al. 2012; Granjou et al. 2013; Soberón and Peterson 2015). Analyses of the disciplinary composition of the Multidisciplinary Expert Panel (MEP) (Montana and Borie 2016; Kovács and Pataki 2016; Morin et al. 2017), as well as of expert groups in charge of the first IPBES assessments (Reuter, Timpte, and Neßhöver 2016; Timpte et al. 2018), reveal an underrepresentation of experts from SSH scholarships, intensifying debates on what is causing it (Vadrot, Jetzkowitz, and Stringer 2016; Reuter, Timpte, and Nesshöver 2016; Rankovic et al. 2016; Stenseke 2016). According to Anne Larigauderie, Stenseke, and Watson (2016), “[a] strong collective effort is necessary to reach scholars outside the natural sciences, because they might not consider themselves to be biodiversity researchers” (Larigauderie, Stenseke, and Watson 2016). At the same time, because early IPBES works and debates have so strongly been framed with questions and categories pertaining to natural sciences, social scientists themselves often “[…] still don't see what role they could play in an institution dedicated to biodiversity” (Roué, Rankovic, and Biagiotti 2017).^1^

The discrepancies between the objective to involve SSH in environmental science and policy and the actual practices of interdisciplinarity persist far beyond IPBES (e.g. Barry and Born 2013; Lahsen 2013; Sandbrook et al. 2013; Victor 2015; Bennett et al. 2017). The quest for interdisciplinarity causes controversies, revealing the asymmetries between scientific disciplines, research traditions, funding organizations and academic institutions and practices (e.g. Barry and Born 2013, Weszkalnys and Barry 2013; Viseu 2015). Viseu (2015) has put her experience into her own words:

Rather than being scientists in our own right, we are brought along as silent partners whose job it is to care for science. Rather than blurring boundaries and labour divisions, integration works to reify them. Thus, the questions that social scientists ask and the expertise we can contribute are muted or made invisible because we remain outside “proper” science. (Viseu 2015, 291)

In order to prevent that current debates and practices over the integration of SSH in IPBES perpetuate these frustrations, this paper proposes a careful analysis of the expectations towards SSH voiced in the literature in the past 11 years. Rather than reflecting on the causes of disciplinary imbalance (Stenseke 2016; Reuter, Timpte, and Nehöver 2016) or justifying the need for further integrating SSH in global environmental knowledge production (Jasanoff 2010; Lövbrand et al. 2015; Bennett et al. 2017) this article will try and work out the baseline from which the role of SSH in IPBES can be strengthened.

The paper departs from the assumption that the contributions of SSH to IPBES can be of very different nature, depending, for example, on the stage at which experts from SSH are involved, but also on the scope and wording of an assessment and its objectives and contents. A systematic analysis of the manifold roles and contributions of SSH could significantly increase the feasibility and effectiveness of measures developed and implemented to ensure that IPBES meets its objectives of multi- and interdisciplinarity. So far, a diverse range of expectations – e.g. how the role(s) of SSH is imagined – seems to have been expressed in the literature on what SSH could do for, and in relation to, IPBES and its current and future work programmes. However, these expectations have not been systematically assessed and discussed.

The aim of this paper is to identify the range of possible SSH contributions to IPBES that are expected in the literature, and discuss the concrete ways to realize these contributions in the particular institutional setting of IPBES. We address these two points by: *firstly*, assessing the literature dealing with IPBES and building a typology describing the main ways in which contributions from SSH to IPBES have been conceived so far. We will discuss these expected contributions in light of broader debates on the role of SSH in nature conservation, discuss how these debates relate to IPBES’ conceptual framework and analyse some of the blind-spots in the perception of how SSH could substantially contribute to the works of IPBES (Section 3.). Then, *secondly*, by looking at one particular example, economics and its use in the assessment on pollinators, pollination and food production, we will concretely illustrate how selective perceptions of the role of SSH contribute to narrowing down our understanding of the drivers and causes of biodiversity loss. The analysis of our case will show how works in a given discipline could contribute in many different ways to the works of IPBES and help identify paths for enhancing the conservation of biodiversity and its sustainable use (Section 4.). *Finally*, we propose a range of practical recommendations as to how to increase the contribution of SSH in the works of IPBES, by following the different procedural steps that lead to the production of a given IPBES assessment (Section 5/conclusion).

## 2. Methodology

**Figure 1.**
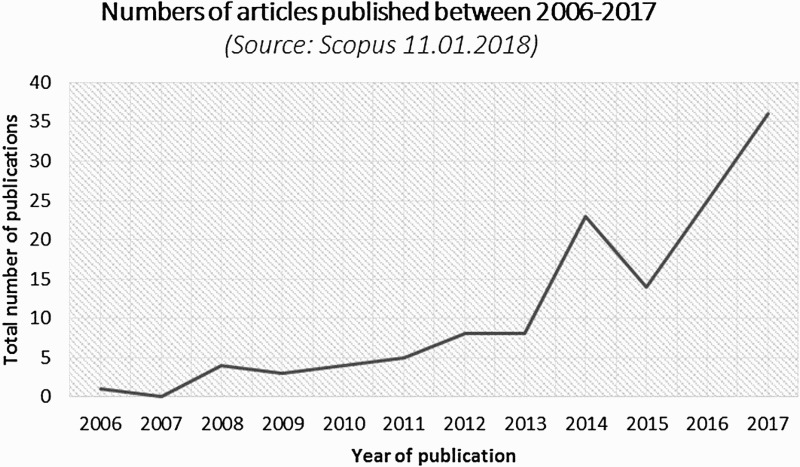
Yearly number of documents published from 2006 to 2017 including the terms IPBES or IMoSEB in their title, abstract or key words. Source: Scopus, search date 11.01.2018

A systematic review of mostly peer-reviewed titles was conducted by following a three-step method. *Firstly*, a research on Scopus was performed with the search query “IPBES or IMoSEB” in their title, abstract or key words.^2^ This yielded a list of 123 papers, published from 2008 to 2017 (as of 11th of January 2018). Figure 1 gives an overview over the number of articles published between 2006 and 2017. An increase in interest in IPBES is visible over the period, particularly after IPBES was successfully established in 2012 and after the first work programme of the platform was released in 2014.

*Secondly*, the 123 identified documents were downloaded. The abstracts were thoroughly read and categorized. Only the papers that explicitly addressed IPBES work or process (e.g. not simply referring to IPBES) were kept in the corpus, which led to 111 papers. The remaining 111 abstracts were screened, looking for mentions, either explicitly or implicitly, of SSH. To further illustrate this point, Stenseke (2016) discusses the obstacles for engaging SSH in IPBES: this was considered as a paper explicitly mentioning the role of SSH in IPBES. The role of SSH in IPBES was considered to be addressed implicitly when, for example, a paper pointed to the need of understanding the social and political structures within which measures to halt the loss of biodiversity are implemented (e.g. Hill et al. 2013; Schmeller, Niemelä, and Bridgewater 2017). Documents discussing or studying IPBES as a “science-policy interface”, without discussing the kind of knowledge relevant to IPBES were excluded (e.g. Brooks, Lamoreux, & Soberón 2014). This second step led to retain 45 papers in the corpus.

*Thirdly*, these 45 papers were inserted into *atlas.ti*, a text analysis software, which provides support to qualitative analysis. Their whole text was examined in search for mentions of SSH and their place, actual or potential, in IPBES works. These mentions were coded to identify and distinguish the expected types of contributions from SSH and build a typology representing the different expectations and areas of intervention of SSH. The process that led to this typology was iterative: while analysing papers, we checked that the mentioned SSH contributions could be unambiguously classified into one of our categories and that there were no missing category nor category overlap. After analysing all the papers, a general double-check was performed.

## 3. Possible contributions of SSH to IPBES: what is expected?

### 3.1. A typology of expected contributions from SSH to IPBES

Based on our analysis, we suggest that discussions over SSH in IPBES are organized on two dimensions: (i) the thematic areas on which SSH can intervene, and (ii) the concrete contribution that they can make in a given thematic area. We distinguish three thematic areas, where SSH is expected to play a role: (1) *Drivers and causes of biodiversity loss*, (2) *Politics and policies for biodiversity conservation and use,* and (3) *IPBES and the politics of knowledge*. In each of these areas, it is considered that SSH can make three types of contributions, by engaging in (a) *Contextualizing and framing*, (b) *Knowledge production*, and (c) *Process design*.

*(1) Drivers of biodiversity loss*. This is a thematic area concerned with understanding the social and human-related processes of biodiversity loss. By drivers, or “indirect drivers”, we mean: the effects of how societies govern themselves and the impact of how political institutions and policies are designed to regulate the protection and use of natural resources (e.g. agricultural policy, spatial planning, or trade). Causes, or “underlying causes”, describe those aspects of human activities deeply entrenched in the political, economic and cultural self-understanding of how land and ecosystems are and should be used, shared and managed (e.g. production, consumption, reproduction, and land use practices). Within papers of our sample, the following aspects fall under drivers or causes: “unsustainable ways of life” (Duraiappah and Rogers 2011, 221), “human well-being” (Duraiappah et al. 2014; Mlambo 2012; Díaz et al. 2015; Larigauderie et al. 2012) and “land use change” (Hill et al. 2013, 41). The emphasis on the role of “human activities” is also central to the MA.

The MA […] engaged natural and social sciences and it focused not only on the status of biodiversity elements but also on their products and functions, specially ecosystem services, or benefits received by society thanks to the functioning of ecosystems. (Larigauderie and Mooney 2010, 10)

*(2) Politics and policies for biodiversity conservation and use.* This thematic area concerns the political frameworks and the range of instruments specifically established to tackle the loss of biodiversity at different policy levels and scales. This thematic area covers the formal institutional settings established to address biodiversity loss at the international scale, such as the *Convention on Biological Diversity* (CBD) or its *Subsidiary Body on Scientific, Technical and Technological Advice* (SBSTTA), and the political conditions for implementing multilateral environmental agreements and national nature conservation policies at the local level. Descriptions linked to this area often refer to the so called “implementation deficit” (Koetz, Farrell, and Bridgewater 2012; Bridgewater 2017) and the “lack of efficiency” in the management of biodiversity and ecosystem services (Balvanera et al. 2014). Another issue concerns the role of conflicts and power struggles, described as central barriers to implement nature conservation measures:

[…] underlying drivers of ecosystem change may be tied to latent conflict among groups. Ecosystem assessments should not ignore the historically-carved interests of actors that manage landscapes nor the struggles and negotiations that have resulted in the current distribution of ecosystem services. (Berbés-Blázquez et al. 2016, 140)

Thus, understanding the nature of institutions and conflicts related to the protection and use of biodiversity at different scales is of great importance for developing appropriate policies.

*(3) IPBES and the Politics of knowledge*. The role of SSH in this area needs to be understood against the background of ongoing debates on how to enhance the uptake of scientific knowledge in nature conservation policy (Rose et al. 2017). Marine ecologist Jane Lubchenco has made the case that the best biodiversity policy and its management are based on the “best science” (Lubchenco 1995) and by doing so evoked the idea of an “IPCC for biodiversity” (Vadrot 2014b). In the past 15 years, several attempts have been made to develop and further improve “science-policy interfaces” for biodiversity and to describe the means by which they could contribute to tackling the perceived lack of scientific advice and consensus in international biodiversity politics (e.g. van den Hove 2007; Koetz et al. 2008; Neßhöver et al. 2013; Neßhöver et al. 2016; Beck et al. 2014; Carmen et al. 2015). Within our sample, emphasis is placed on the design of science-policy interfaces and the conditions for participation and pluralism. This also includes the forms of (non-academic) knowledge needed to tackle biodiversity loss and the institutional and epistemic conditions for inter- and transdisciplinary approaches and assessment work.

In each of these areas, it is considered that SSH can make three types of contributions:

- *Contextualizing and framing.* SSH perspectives can contribute to the creation of new forms of understanding and communicating biodiversity loss within and beyond political and institutional frameworks within which nature conservation policies and measures are developed and implemented.
- *Knowledge production*. SSH are considered to play an important role in producing new knowledge and research in the three areas identified above.^3^
- *Process design*. By process design we mean the evaluation, development and implementation of new concepts, instruments and methodologies for improving research approaches, policy-making, and producing fruitful interactions between actors.

Crossing the three types of thematic areas and contributions yields a matrix of 9 different expected contributions from SSH to IPBES (see Table 1). Below, we describe these expected contributions, grouping them by thematic area.

**Table 1.** Typology of expected contributions from SSH to IPBES.

		Thematic area		
		(1) Drivers and causes of biodiversity loss	(2) Politics and policies for biodiversity conservation and use	(3) IPBES and the politics of knowledge
**Types of contributions from the social sciences and humanities**	**(a) Contexualization & framing**	Understanding socio-ecological systems and how they relate to human well-being, human rights, equity and justice	Understanding the role of values, institutions, conflicts and power relations in policy-making and implementation	Understanding the nature of knowledge and how science-policy interfaces work
	**(b) Knowledge production**	Examining drivers and causes of environmental and societal change and explaining how human behaviour, values, narratives and worldviews can be changed	Examining the effectiveness of institutions and policy and economic instruments	Examining barriers and opportunities for participatory knowledge production and pluralistic methodologies and epistemologies
	**(c) Process design**	Developing indicators and approaches for mapping, comparing, and predicting practices of biodiversity conservation and use	Developing techniques for deliberation, participation and pluralistic (valuation) approaches for modelling, predicting and assessing risks, trade-offs and conflicts	Developing concepts and instruments for designing and operating science-policy interfaces according to the principles of inter- and transdisciplinarity, participation and co-production

#### 3.1.1. Drivers and causes of biodiversity loss

*(1a) Understanding socio-ecological systems and how they relate to human well-being, human rights, equity and justice.* This type of expectation relates to the assumption that understanding the drivers and causes of biodiversity loss necessitates that nature and society are conceptualized as inherently interdependent and forming “socio-ecological systems” (e.g. Díaz et al. 2015; Pascual et al. 2017). In this respect, SSH are conceived as important sources for framing biodiversity issues as not only “purely natural” phenomena, e.g. in terms of species extinction rates. Instead, it is argued, the way we talk about and address biodiversity conservation and its sustainable use must acknowledge the multiple human dimensions of these issues (political, economic, institutional, ethical, etc.) (Hill et al. 2013; Brand and Vadrot 2013; Vadrot 2014b; Bridgewater 2017). This is deemed important to better address issues of human well-being, human rights, equity and justice associated with biodiversity conservation and use, as well as to be able to identify the root causes of biodiversity loss (see next point).

*(1b) Examining drivers and causes of environmental and societal change and explaining how human behaviour, values, narratives and worldviews can be changed.* The notion of change is mentioned often in relation to the forms of knowledge needed, particularly with regard to human behaviour and the kinds of worldviews, values, and beliefs influencing social practices, especially when considered unsustainable (e.g. Duraiappah and Rogers 2011; Larigauderie et al. 2012; Berbés-Blázquez, González, and Pascual 2016). Duraiappah and Rogers (2011) argue that basic research is needed to identify how values change and how particular incentives can be used to contribute to this substantive change. Individual behaviour and institutions are conceived as important drivers of biodiversity loss, but also as targets for initiating changes in the values, narratives and worldviews underpinning unsustainable practices and habits. Hence, what prevents and what enables change are described as a major contribution of SSH to the works of IPBES (Berbés-Blázquez, González, and Pascual 2016; Pascual et al. 2017).

*(1c) Developing indicators and approaches for mapping, comparing, and predicting practices of biodiversity conservation and use.* Here, the perceived role of SSH is to contribute to the development of approaches and concepts to systematically study past and present practices of biodiversity conservation and use. In line with ecological research examining patterns in ecosystem change on the basis of “biodiversity indicators” or “Essential Biodiversity Variables”, SSH is expected to complement existing approaches and data sets with indicators covering socio-economic aspects of biodiversity loss. This includes data from national statistical bureaus (Geijzendorffer et al. 2017) and the development of socio-economic models and scenarios (Larigauderie and Mooney 2010; Kok et al. 2017). Thus, indicators and socio-economic scenarios are important pillars for representing social aspects in prediction exercises. More specially, some authors propose commodity chain analyses and historical mapping approaches (Berbés-Blázquez, González, and Pascual 2016) to increase our understanding of behavioural and institutional patterns of past and present unsustainable practices causing biodiversity loss.

#### 3.1.2. Politics and policies for biodiversity conservation and use

*(2a) Understanding the role of values, institutions, conflicts and power relations in policy-making and implementation.* The recognition of the inherently normative character of biodiversity conservation practices in different parts of the world is described as a precondition, which needs to be taken into account in debates over the development of more effective policies (e.g. Duraiappah and Rogers 2011; Stenseke 2016; Pascual et al. 2017; Laurans 2018). The nature of conflicts and power relations, particularly with respect to the concept of ecosystem services, is often described as an important, but underrepresented aspect of the politics of conservation (Stenseke 2016; Brand and Vadrot 2013; Vadrot 2014b). Different values associated with biodiversity and its use challenge the development of governance structures and institutions designed to protect nature (Díaz et al. 2015; Pascual et al. 2017). Consequently, the recognition of political, socio-economic and legal issues could contribute to increase the acceptance of conservation politics and policies, particularly with regard to the context of the CBD and its SBSTTA (Koetz, Farrell and Bridgewater 2012; Bridgewater 2017). Some authors criticize the narrow framing of biodiversity and related policies. Stenseke (2016) suggests that concepts such as “landscape”, “driving forces”, and “livelihoods” could enrich the natural science focus. Bridgewater (2017) argues that the role of heritage and the notion of biocultural diversity should increase in importance within IPBES related conceptualizations.^4^

*(2b) Examining the effectiveness of institutions and policy and economic instruments.* This type of expected contribution from SSH is closely tied to the assumption that some of the difficulties identified in 2a could somehow be solved. Examining the effectiveness of institutions involved in biodiversity policy-making is one field. The other field covers issues linked to the effects and the impact of the biodiversity conservation measures themselves. Here, SSH are expected to contribute to measuring the costs of inaction, understanding trade-offs, and bridging values and different value systems (Díaz et al. 2015; Pascual et al. 2017; Bridgewater 2017). Another task which SSH could perform is the conceptualization of power in ecosystem services management (Berbés-Blázquez, González, and Pascual 2016) and the examination of the link between economic incentives and behaviour (Duraiappah and Rogers 2011; Díaz et al. 2015). Understanding the role of economic incentives, taxations and subsidies more generally, is described as important contribution to increasing the positive effects of nature conservation policy and tackling related trade-offs with other land-uses.

(2c) Develop techniques for deliberation, participation and pluralistic (valuation) approaches for modelling, predicting and assessing risk. The contributions of SSH to process design are divided into three categories mentioned within the documents of our sample. *Firstly*, approaches and techniques to value biodiversity and ecosystem services. *Secondly*, approaches to predict or identify policy options such as the Delphi technique (Tengö et al. 2014), multi-criteria analysis (Berbés-Blázquez, González, and Pascual 2016), risk assessments and socio-economic scenarios and models (Larigauderie and Mooney 2010; Laurans 2018). *Thirdly*, methods to ensure participation and ownership such as participatory scenarios analysis (Berbés-Blázquez, González, and Pascual 2016), scenario building (Stenseke 2016), deliberative policy tools (Pascual et al. 2017) and concepts for mediating, organizing and facilitating interfaces between different actor groups and fields (Carmen et al. 2015; Löfmarck and Lidskog 2017; Laurans 2018).

#### 3.1.3. IPBES and the politics of knowledge

The necessity to involve SSH in the processes and products of biodiversity related science-policy interfacing mechanisms was recognized and discussed very early in the debate over establishing IPBES (e.g. van den Hove 2007; van den Hove and Chabason 2009; Koetz et al. 2008; Duraiappah and Rogers 2011; Larigauderie et al. 2012; Vadrot 2011). In the following, the three different types of roles attributed to SSH with regard to this third thematic area will be described.

*(3a) Understanding the nature of knowledge and how science-policy interfaces work.* Contextualizing and framing the relations between science and policy as complex and interdependent is conceived as a condition for ensuring the production of policy relevant biodiversity knowledge. The same is true for the nature of knowledge itself and the recognition of diversity within different knowledge systems (Díaz et al. 2015; Pascual et al. 2017). The description of science-policy interfaces as social processes, as described by van den Hove (2007), is often used as an entry point for describing how SSH helps to think beyond the so-called “linear model of expertise” (e.g. Koetz, Farrell, and Bridgewater 2012; Neßhöver et al. 2013; Neßhöver et al. 2016; Livoreil et al. 2016; Beck, Esguerra, and Goerg 2017). Furthermore, STS concepts are conceived as means by which epistemological barriers and disciplinary boundaries can be contextualized and challenges in the production of interdisciplinary knowledge in and beyond IPBES addressed (Obermeister 2017; Montana 2017). Thus, the role of SSH in framing and contextualizing is closely linked to the perception of conflicts and challenges in bridging different knowledge forms and creating related institutional spaces.

*(3b) Examining barriers and opportunities for participatory knowledge production and pluralistic methodologies and epistemologies.* The research to be conducted in this area should mainly address the conditions for participatory knowledge production and pluralism. This includes knowledge on the institutions more generally and instruments for participation in knowledge production practices (Neßhöver et al. 2013; Carmen et al. 2015; Tengö et al. 2014, 2017) Furthermore, SSH can play a role in addressing, analyzing and solving conflicts emerging within and beyond IPBES, for example, “when attempting to get physical and natural scientists to accept the inherent plurality and locality of knowledge […]” (Obermeister 2017, 85). Conflicts related to knowledge pluralism are described in relation to the differences within types of scientific knowledge and forms of evidence, such as between disciplines of social and natural sciences or quantitative and qualitative approaches (Tengö et al. 2014, 580; Stenseke 2016; Hotes and Opgenoorth 2014; Laurans 2018).This aspect relates to the claim that the benefits and contributions of qualitative research and how they are linked to, and can be combined with, quantitative methodologies need to be examined more closely (e.g. Tengö et al. 2014; Berbés-Blázquez, González, and Pascual 2016; Stenseke 2016; Löfmark and Lidskog 2017).

*(3c) Developing concepts and instruments for designing and operating science-policy interfaces according to the principles of inter- and transdisciplinarity, participation and co-production*. Process design in this area is strongly related to the role of SSH in the development and implementation of science-policy interrelations and mechanisms to overcome the challenges described in 3a) and 3b). Science-policy interfacing mechanisms are often described as complex and resource intensive activities, which remain underestimated and undervalued in most research and policy contexts (Neßhöver et al. 2013, 102). The engagement with the practical, theoretical and ethical conditions of making such mechanisms work, is thus a main issue of concern, particularly with regard to the principles of inter- and transdisciplinarity and new forms of participation and stakeholder engagement (e.g. Arpin et al. 2016; Esguerra, Beck, and Lidskog 2017; Tremblay, Vandewalle, and Wittmer 2016; Oubenal, Hrabanski, and Pesche 2017). Tengö et al. (2014) for example call for the development of a new approach, which they name the “Multiple Evidence base approach” particularly suitable for “local to global knowledge-policy processes such as IPBES or the SDGs” (Tengö et al. 2014, 580). Esguerra, Beck, and Lidskog (2017) address the role of consensus in stakeholder participation in IPBES and Neßhöver et al. (2016) discuss the integration of science-policy interfaces within research projects and call for a broader recognition of the engagement of scientists within processes located at the intersection between scientific research and policy-making. The evaluation of existing mechanisms, such as IPBES and the design of new approaches for facilitating co-production of different scientific disciplines, knowledge forms and methodologies is a role attributed to SSH in this context.

### 3.2. Discussing the typology and identification of shortcomings

Our typology aimed at identifying different expectations towards the contribution of SSH to IPBES. On this basis we aimed at proving a differentiated view on the roles and areas of SSH interventions. In the following, we will relate our analysis to discussions of what SSH can contribute to nature conservation more generally and show why our typology is important to identify blind spots and be more precise in the formulation of future research questions and knowledge needs.

For at least two decades ecologists have agreed on the relevance of the social sciences and humanities in and for nature conservation (Sandbrook et al. 2013). The recognition that conservation is not solely about species or ecosystems, but about people, has solidified debates among nature conservationists on the kinds of social research needed to support conservation efforts (Mascia et al. 2003, 649):

Political science, anthropology, economics, psychology, sociology, geography, legal studies, and other social science disciplines all have analytic tools and established knowledge that can explain and predict patterns of human behavior – insights vital to the success of local, national, and international conservation efforts. (Mascia et al. 2003, 649)

Hence, the main question was – and as our analysis confirms – not if, but rather how to integrate or mainstream the social sciences in and for biodiversity conservation (e.g. Barry and Born 2013; Bennett et al. 2017).

Sandbrook et al. (2013) argue that interdisciplinary research still suffers from a misunderstanding among nature conservation scientists on what conservation social science is (Sandbrook et al. 2013, 1487). The authors argue that severe communication problems between the natural and the social sciences continue to hamper interdisciplinary research, regardless the many efforts that have been made in developing solutions within the conservation community itself (e.g. Campbell 2005; Brosius 2006; Fox et al. 2006). For the sake of clarification, Sandbrook et al. (2013) differentiate between social research *for* conservation and social research *on* conservation. Social research for conservation explicitly aims to contribute to halting the loss of biodiversity by identifying societal causes, or “indirect drivers” – in IPBES terminology – leading to the destruction of ecosystems and biodiversity (Sandbrook et al. 2013, 1487). In comparison, social research on conservation is interested in the institutions, discourses, and practices constituting the conservation movement and its agents itself (Sandbrook et al. 2013, 1488). The authors admit that the distinction between the two is not always clear-cut. For example, they assume that social research for conservation shares with conservation biology the objective to halt the loss of biodiversity, which might not necessarily be the case for social research on conservation. However, this does not mean that social researchers on nature conservation in general have no interest in the protection of biodiversity. When we look at the typical research questions Sandbrook et al. (2013) associate with the two modes of social research, their distinction becomes clearer: Social research for conservation would for example ask: “what socioeconomic factors contribute to effective conservation strategies and get support from society, and how to get society to support conservation policies”. In turn, research on conservation is interested in “what conservation reveals about the broader political economics structures and power relations of which it is part, and the way in which these influence human-nature relation” (Sandbrook et al. 2013, 1488).

A closer look at the conceptual framework (CF) of IPBES, indeed, reveals that both modes of social research are conceived as important contributions to IPBES assessments. The CF refers to knowledge needs in the areas of “institutions and governance and other indirect drivers”, “anthropogenic assets” or “anthropogenic drivers”. “Anthropogenic assets” for example are “built infrastructure, health facilities, knowledge (including ILK and technical or scientific knowledge, as well as formal and nonformal education), technology (both physical objects and procedures), and financial assets, among others” (Díaz et al. 2015, 14). Understanding for example how the implementation of new nature conservation policies in a particular area in Madagascar, such as the introduction of eco-tourism initiatives or fair trade of local food, influences the way in which a community manages and values a particular piece of land, and how this impacts on biodiversity, would require research on the practices, routines and value systems of this particular community and the social and political orders characterizing it. This example shows that social research on and for biodiversity conservation are also overlapping and increasingly intertwined.

Our typology confirms that the two types of social research described by Sandbrook et al. (2013) and addressed in the CF indeed interrelate. “Indirect drivers” of biodiversity loss refer to the effects of how societies govern themselves and the impact of how political institutions and policies are designed, which also includes biodiversity conservation policies and practices. However, these can only be properly analysed and evaluated if they are linked to the societal frameworks within which they are developed and implemented. Analysing those aspects of human activities, deeply entrenched in the political, economic and cultural self-understanding of how land and ecosystems are and should be used, shared and managed, is a pre-condition for assessing the causes of biodiversity loss. This, however, also and often includes the nature conservation practices themselves (e.g. Büscher et al. 2017).

Distinguishing between the different roles and areas allows us to identify blind spots and be more precise in the formulation of future research questions and knowledge needs. The questions what the area of intervention is and what kind of contribution can be made, cannot be asked independent from each other. The lack of clarity on how different types of contributions relate to the areas of intervention, bears the danger of inadequacies in the understanding of what the social sciences and humanities can contribute. In particular, we identify the following problems, which we relate to the three roles of SSH:

*Contextualizing and framing*: SSH perspectives can, indeed, contribute to the creation of new forms of understanding and communicating biodiversity loss and in relation to policies, politics, and co-production. However, there is a danger that the “social science and humanities perspective” is seen as ONE particular perspective, which could or should be added to already existing framings of empirical evidence and ways of contextualizing the three areas we have identified (e.g. Viseu 2015; Barry and Born 2013). Instead, there is a diversity of framings within different SSH disciplines, theories, and research areas. Depending on the social theory, or in other words, the paradigms and analytical frameworks used, social phenomena are examined and explained differently. This includes ideas about societal change, behaviour, power or political and social structures. How conflicts related to land use or trade-offs between nature conservation and other economic activities are explained and contextualized heavily depends on the theoretical and normative assumptions of a given social scientific approach. Neglecting this diversity and normativity, and formulating the contribution of SSH against very particular understandings of power and change, bears the danger that particular approaches will not be heard and others systematically reproduced (e.g. behaviourist approaches, actor-centred understandings of change, hierarchical understandings of power, neoclassical economics, etc.). Vadrot (2014a, 2017) has described the underlying mechanisms and dynamics as relational processes, whereby “epistemic selectivities”, structuring the field within which actors formulate their interests, are both strategically and unconsciously reproduced by those actors to increase the likeliness that their positons will be heard.

*Knowledge production*: SSH are considered to play an important role in producing new knowledge and in contributing to inter- and transdisciplinary research in the three areas. Our typology refers to three areas, where SSH is expected to contribute. In all three cases the descriptions are solution-oriented and driven by the general view that examining drivers, causes, barriers and opportunities could contribute to change, effectiveness and efficiency. Whilst there is, more generally, nothing wrong with this perspective, there remains the danger that the kinds of questions asked are formulated against the background of a narrow understanding of particular social phenomena. A political scientist working within the paradigm of institutionalism is more likely to address the efficiency of institutions to increase the effectiveness of nature conservation policies than a political ecologist, who would rather look at the effects of an institution on the distribution of the costs and benefits related to environmental change within a particular community (Bryant 2015; Peet, Robbins, and Watts 2011). The same is true for economics, where an analyst focused on cost–benefit analysis to reveal the hidden costs of biodiversity loss, for instance, will not produce the same conclusions than an economist using economic valuation as a way to reveal the diversity of material attachments – and thus value and power conflicts – associated to certain land-use changes (Laurans 2018). Furthermore, and as suggested by Viseu (2015) there is a danger to view SSH knowledge as complementing research framed and developed by natural scientists, or simply as an additional asset to natural science findings for better implementation and communication at a later stage.

*Process design*: Certainly, and, as we have shown, SSH are expected to contribute to the evaluation, development and implementation of new concepts, instruments and methodologies for improving research approaches, policy-making, and producing fruitful interactions between actors. Process design, however, depends on the goals to be attained. Furthermore, the appropriateness of instruments and approaches related to the economic, political and cultural context within which they are designed and applied. Whilst the role of cultural, national and political frameworks is generally acknowledged, the need for specific SSH knowledge taking these aspects into account at the level of process design, is not always made explicit. For example, the practices of political participation and the role of expertise and evidence more generally, strongly depend on the political system and the political culture in a nation state, in different regions, in an organization or a community. The degree to which citizens accept and make use of participatory approaches and democratic instruments (e.g. citizen’s initiatives or participatory urban planning), strongly depends on the political culture of a country or region, not to mention the existence of strong institutional and legal structures within which such processes can adequately be run and implemented. Sheila Jasanoff (2007) has developed the concept of “civic epistemologies” to describe the culturally specific ways in which publics expect the state to use expertise, knowledge, and reasoning in decision-making in a very particular way. The same is true for the participation of SSH experts in IPBES assessments. The rules and procedures organizing the nomination of IPBES experts by nation states strongly differ and challenge the development of top-down solutions (Timpte et al. 2018).

In the following, we will illustrate how a selective understanding and use of SSH in IPBES, contributes to weaken the potential explanatory power of IPBES assessments. By using the example of economics and how it has been used in the pollination assessment, we aim to exemplarily show, how selective understandings of a discipline contribute to the maintenance of narrow approaches to biodiversity politics, *inter alia* mirrored in controversial debates over the “neoliberalisation of nature” (e.g. Turnhout, Neves and Lijster 2014; Apostolopoulou and Adams 2016; Holmes and Cavanagh 2016; Büscher et al. 2017). In this respect, economics constitutes an illustrative example of the alternate ways in which SSH can contribute to the works of IPBES.

## 4. One discipline, many possibilities: economics and pollination as an example

The activities of IPBES started in a period marked by the omnipresence of economic approaches to biodiversity issues (e.g. the ecosystem service framework, widely influenced by welfare economics theory, or The Economics of Ecosystems and Biodiversity – TEEB – initiative). In this context, several parties to IPBES expressed concerns that economic approaches, such as monetary valuations, would be the only way used to address human dimensions of biodiversity issues in IPBES works. This was well illustrated in the debates surrounding IPBES’ conceptual framework, where terms such as “ecosystem services” were rejected by some countries because it was said to represent hegemonic, Western, economic approaches to nature (Brand and Vadrot 2013; Vadrot 2014a, 2014b; Borie and Hulme 2015).

These debates, however, tend to reduce economics to a rather narrow function, namely a role of ecosystem service valuation (ESV). This is, for instance, mostly how economics are used in the assessment on pollinators, pollination and food security (in its chapter 4). In this approach, economics serves to “reveal” the importance of biodiversity for human activities, or, as TEEB put it, to “make nature’s values visible”.^5^ The underlying assumption is that biodiversity loss is mostly due to a lack of understanding of its economic importance and that monetary valuations could demonstrate, especially to decision-makers, that conserving biodiversity is the optimal collective option (Laurans and Mermet 2014).

Counting on economics primarily for valuing ecosystem services leads to two different kinds of problems. *Firstly*, the importance of ESV contribution to actual decision-making is not demonstrated empirically (Laurans et al. 2013), despite authoritative claims (e.g. National Research Council 2005). *Secondly*, it prevents the development of other and potentially fruitful contributions of economics to IPBES. Indeed, economics is not necessarily confined to demonstrating the importance of nature conservation. In a context where multiple commitments have already been made, at all levels, to stop biodiversity loss, a major contribution to IPBES from economics in the coming years could be assessing and discussing the economic justification of contrary forces that hamper the implementation of biodiversity policies and the fulfilment of conservation objectives. Here, economics could play a major role in revealing, not “the hidden value of nature” but, rather, the social phenomena that materially link human actions and biodiversity loss. In what follows, this will be exemplified through (i) the contribution of economics to public policy analyses and the identification of subsidies, or public incentives, that are harmful to biodiversity and (ii) the analysis of global value chains to understand where major economic pressures on biodiversity come from and who has the most power to act upon them.

Addressing the underlying causes of biodiversity loss constitutes the Strategic Goal A within the Aichi Biodiversity Targets established in 2010. Underlying causes of biodiversity loss, in terms of incentives that are harmful to biodiversity, must therefore be better investigated and understood if one is to achieve Aichi target 3 and implement decision XII/3 on Resource mobilization adopted at the 12th COP of the CBD in 2014. Importantly, economics, within IPBES assessments and discussions, can usefully contribute to this by (among others): (1) screening economic activities linked to deteriorating environmental values; (2) discussing and assessing the collective profitability of projects, plans and policies that result in biodiversity erosion; (3) enlightening the scope of economic and technical options that would allow for carrying out economic activities while minimizing their impact on biodiversity; (4) establishing a list of all public subsidies in a given country which influence these economic activities and thus impact on the status biodiversity and is component, and discussing their economic rationale; (5) attempting to describe certain causal links between those public subsidies and the loss of biodiversity; (6) quantifying these subsidies; and (7) assessing the environmental benefits of removing environmentally harmful subsidies.

Building on previous work (OECD 2005; TEEB 2009; Valsecchi et al. 2009), Sainteny et al. (2015) analyse public incentives that are harmful to biodiversity in France. Led by two economists, the public report allows to precisely assess how harmful subsidies contribute to driving biodiversity loss and thus how much their phasing-out would be beneficial to biodiversity. For instance, by contributing to urban sprawl, incentives to purchase land for residences encourage the destruction of natural habitat. Exemptions from the domestic consumption tax on oil products contribute to overfishing, while laws or taxes on industry and transportation (and their exemptions) exacerbate water and air pollution issues. In this context, a political economy approach could provide IPBES with tools to grasp the complexity of public policies, reduce hurdles, define trade-offs and analyse strategies from interest groups, winners, loosers and economic explanations for status quo. In fine, the thorough evaluation of public policies, in a broad perspective, would help agreeing on operational recommendations for economically realistic and feasible actions to curb biodiversity loss.

Economic analysis might further help parties to IPBES in disentangling mechanisms at play, and their critical impacts on biodiversity, in the production, exchange and consumption of goods and services at the local as well as global levels. Parties to the CBD (at COP12 and COP13) broadly acknowledged the need to understand in depth the contribution of “economic sectors” to biodiversity erosion (Kok et al. 2014) and thereafter push for sustainable production and consumption (e.g. decision XII/ 10 at COP12, see UNEP/CBD/COP/DEC/XII/10). One way, it is contented, is to study supply chains (Recommendation 1/4 from the SBI, 2016; Kok et al. 2014), alternatively referred to as “value chains”, which connect, increasingly at the global level, producers of commodities, traders, transforming companies, branders and distributors along a chain of material, contractual and financial relations. On the one hand, economists contribute to demonstrate the factual causal links between international trade along those global chains and deforestation (Persson, Henders, and Kastner 2014) as well as threats to species (Lenzen et al. 2012) at the local level. On the other hand, a global value chain approach (Gereffi and Korzeniewicz 1994; Gereffi, Humphrey, and Sturgeon 2005), a multidisciplinary combination of political economy, institutional economics and sociology, can help to inform the governance of such chains at the global level. Understanding the underlying causes (Strategic Goal A within the Aichi Biodiversity Targets) and direct pressures (Strategic Goal B) could thus be improved by studying power plays across economic actors within supply chains, contractual and institutional arrangements between them, and price-setting mechanisms. These factors can explain an important part of production practices, how agrarian systems are organized, and their role in biodiversity loss. Through assessing such works, IPBES experts could assess the impacts of production and consumption patterns, and their current organization, on biodiversity (Bolwig et al. 2010), and propose meaningful regulatory and market-based solutions (Newton, Agrawal, and Wollenberg 2013). These could include certification or voluntary agreements (such as FLEGT, “Forest Law Enforcement for Governance and Trade”), to “clean” commodity supply chains.

In the pollination assessment, as said above, economics have been restricted to a very narrow function. Including works on the socio-economic drivers of current agricultural models could have enabled authors to produce recommendations more tightly linked to changing these drivers (Rankovic et al. 2016). More recently, Díaz et al. 2018) acknowledged that,

the ecosystem services research program proceeded largely without benefiting from insights and tools in social sciences and humanities, […] In addition, as diverse disciplines and stakeholders remained at the margins, the initial skepticism toward the ecosystem services framework turned into active opposition, often based on the perceived risks of commodification of nature and associated social equity concerns. (Díaz et al. 2018, 271)

## 5. Conclusion: pathways for a stronger inclusion of SSH in IPBES works

IPBES faces a critical moment in its history (Hof et al. 2017). The first work programme of the platform, which encompassed the first thematic and methodological assessments, four regional assessments and the global assessment, is nearly terminated (albeit with three thematic assessments still pending as we write these lines). Reflections started on how this rather young intergovernmental body will develop and continue the different tasks throughout its second work programme. How should this second work programme be designed and what are the opportunities for the further integration of the social sciences and humanities?

The current shift from “ecosystems to contributions” induced by the introduction of the concept of “Nature’s contributions to people”, as described in Díaz et al. (2018), has reinvigorated the need to discuss the role of the social sciences and humanities in biodiversity science and politics more deeply. However, the realization of interdisciplinarity in and beyond nature conservation constitutes in itself a challenging task, accompanied by asymmetries in the ways in which the natural sciences and SSH are perceived and valued (Barry and Born 2013; Lahsen 2013; Viseu 2015). Ensuring interdisciplinarity within assessment producing bodies such as IPBES (or the IPCC) is further complicated by the highly regulated and institutionalized forms of global knowledge production and its legitimization by nation states in the framework of multilateral negotiations (Hughes 2015; Vadrot 2014a; Montana 2017). The challenges commence with, and are visible throughout, the nomination and selection of experts. The nomination of experts by parties first depends on national priorities and scientific and political cultures. The selection of experts – independent of their disciplinary background – is always political, reflecting individual or collective perceptions and preferences over the way in which nature is represented, governed and used, and the most legitimate knowledge to be represented in the works of IPBES. At the same time, increasing the number of SSH experts in IPBES does not automatically lead to more inter- or multidisciplinarity among the groups of experts involved in the production of IPBES assessments.

Even if one of the primary objectives of IPBES is to assess existing knowledge by selecting recognized experts from all over the world, the assessments themselves produce a new corpus of knowledge linking diverse scientific facts and figures to local case studies and value systems in unprecedented ways. This task, which requires the voluntary work of hundreds of experts is a challenging endeavour, particularly if it is meant to follow the ideal of multi- and transdisciplinarity as suggested by the conceptual framework of IPBES. The diversity of methods and theories employed to analyse the drivers of biodiversity loss are as diverse within the natural sciences as they are, for example, among experts from SSH studying the effects of environmental degradation on local communities and the distribution of costs and benefits. This diversity, which is explicitly acknowledged and demanded by IPBES, constitutes its uniqueness, but is at the same time a trouble spot.

The aim of our analysis was to show that the contributions through which SSH can make a real difference for nature conservation in general and an assessment producing mechanism in particular, are more diverse than assumed, for example by Sandbrook et al. (2013). Instead, we propose a differentiation along *types of contribution* and *thematic areas,* which facilitates our analysis of the kinds of expectations and the epistemic selectivities in place (Brand and Vadrot 2013; Vadrot 2014a, 2017) in place. As we have shown, there is a need to think reflectively and critically about how we frame the role of SSH and to acknowledge the diversity of theoretical and methodological approaches within different disciplines and scholarships, including the normative standpoints from which they depart.

Translated to the needs for IPBES, this means that diverse experts from the fields of SSH should be represented in and involved at different stages of IPBES and its assessments. This conclusion is supported by the results of a workshop at the University of Cambridge, held on the 19th of December 2017, where potential recommendations on how to increase the up-take of SSH knowledge was discussed (Rankovic et al. 2018). In line with the findings of the workshop, we recommend that similar to the task force on local and indigenous knowledge, outlined in deliverable 1 c), IPBES could support the establishment of a task force on SSH. The aim of such as task force could be to identify and discuss *procedures, approaches and participatory processes for integrating the social sciences and humanities*. Furthermore, the task force could act as a facilitator and support the Multidisciplinary Expert Panel in the selection of authors and reviewers for assessment chapters. The group of people involved in the task force, should represent different social science disciplines and methodological and theoretical orientations. They could act as contact points between IPBES and relevant associations and academic societies representing the social sciences and humanities scholarships. By doing so, the task force could significantly support existing efforts of the IPBES secretariat in reaching out to social science communities as exemplified in Larigauderie, Stenseke, and Watson (2016). Additionally, the task force could, similar to the task force on “Knowledge and data”, *prioritize knowledge and data needs for policymaking addressed through catalyzing efforts to generate new knowledge and networking* within the different disciplines and communities of the social sciences and humanities.

Such efforts would significantly support the involvement of SSH experts and their inclusion at different stages of the assessment production procedure, including the process of scoping an assessment. Involving SSH experts at this early stage allows them to contribute more directly to the identification of issue areas and knowledge needs to be covered in different chapters of an assessment. This will increase the resonance of IPBES calls within different SSH communities and facilitate the recognition of the diversity of existing approaches and tools, as we have exemplified with the case of economics and its use in the pollination assessment.

There is a need to avoid that SSH are viewed as attachments or add-ons, which could – in bodies such as in IPBES – be easily represented by natural scientists. A recent contribution published in *Conservation Biology* voices the “[…] hope that the emphasis on disciplinary rigor will also help address the misconception that any biologist can do a social survey or that personal musings constitute a rigorous exploration into the human domain of conservation” (Teel et al. 2018). Whilst in principle such a perspective underlines our claims, the needs for methodological innovations to ensure inter- and transdisciplinarity – for which IPBES could potentially be a testing ground – should not be forgotten.

## Notes

1
Lundquist et al. (2015) have also noticed difficulties in engaging the nature conservation community, which points to more general challenges of attracting experts from academia and practice into processes and products of intergovernmental assessment producing bodies, including awareness, access, capacity, personal resources, perceived benefits and personal values. As Díaz et al. (2018) have pointed out, the early conceptual framework of IPBES and its emphasis on ecosystem services have also discouraged experts not in line with this approach from participating actively in IPBES activities.

2 The consultation process towards an International Mechanism of Scientific Expertise on Biodiversity (IMoSEB) was financed by the French government and ran from February 2006 to November 2007. “These consultations should be seized as a unique opportunity to move biodiversity science and governance forward and to find new ways of resolving the crisis” (Loreau et al. 2006, 246). In 2008 IMoSEB and the MA follow-up were merged and the negotiations on establishing IPBES started under the auspices of UNEP (Vadrot 2014b).

3 In many papers of our sample, knowledge gaps are identified where SSH research or SSH contributions to inter- and transdisciplinary research, are conceived as indispensable contributions for increasing the relevance and applicability of biodiversity research more generally.

4 The attention given to culture “in defining all links between people and nature” has recently be reinforced through the concept of “Nature’s contributions to people”, which “NCP elevates, emphasizes, and operationalizes the role of indigenous and local knowledge in understanding nature's contribution to people.” (Díaz et al. 2018, 270)

5
http://www.teebweb.org.
